# Succinate/IL-1β Signaling Axis Promotes the Inflammatory Progression of Endothelial and Exacerbates Atherosclerosis

**DOI:** 10.3389/fimmu.2022.817572

**Published:** 2022-02-22

**Authors:** Jingwen Xu, Yabing Zheng, Yaqing Zhao, Yujiao Zhang, Huilin Li, An Zhang, Xuehan Wang, Weizong Wang, Yinglong Hou, Jiangrong Wang

**Affiliations:** ^1^Department of Cardiology, The First Affiliated Hospital of Shandong First Medical University and Shandong Provincial Qianfoshan Hospital, Jinan, China; ^2^College of Second Clinical Medicine, Shandong University of Traditional Chinese Medicine, Jinan, China; ^3^Cheeloo College of Medicine, Shandong University, Jinan, China

**Keywords:** atherosclerosis, succinate, Sucnr1, Hif-1α, IL-1β, inflammation

## Abstract

Inflammation is an important driver of atherosclerosis. Succinate is a new extracellular inflammatory alarm released by activated macrophages. Succinate is sensed by succinate receptor 1 (Sucnr1) and then transferred to effector cells. It is worth exploring whether succinate is capable of facilitating the inflammatory response in atherosclerosis. In this study, we firstly found that arterial serum of Coronary Heart Disease (CHD) patients contained significantly higher succinate and interleukin (IL)-1β than Health control (HC) subjects, and succinate was positively correlated with IL-1β. As demonstrated by the *in vitro* study, succinate/hypoxia-inducible factor 1α (Hif)-1α/IL-1β signal axis existed and significantly facilitated the inflammatory program in human umbilical vein endothelial cells (HUVECs). Under the coculture, activated macrophages released succinate, which would be transferred to HUVECs *via* Sucnr1 and then activate Hif-1α to produce a greater amount of IL-1β. Likewise, the aortic sinus’s inflammatory phenotype was found to be more significant within Apoe^-/-^ mice that were injected with succinate. Furthermore, Sucnr1 inhibitor (NF-56-EJ40) could significantly interrupt succinate/IL-1β signal in HUVECs and macrophages. As revealed by this study, glycolytic metabolism following the release of succinate could be found in atherosclerotic pathology, and succinate would drive succinate/IL-1β signal dependent on Sucnr1 and then exacerbate inflammatory responses. Sucnr1 might be a novel target for cutting off the transduction of succinate signal to prevent the inflammation of atherosclerosis.

## Introduction

Atherosclerosis has been reported as the underlying pathology of cardiovascular diseases, which can be significantly driven by inflammation (1). Macrophage deposition and endothelial activation are recognized as the preconditions for arterial inflammation (2). When blood vessels are invaded or injured, macrophages deposit and convert their metabolism from oxidative phosphorylation to aerobic glycolysis (e.g., Warburg effect) after the activation using surface receptors (TLRs, toll-like receptors), which underpins the rapid activation of macrophages and the synthesis of immune mediators required for perpetuating inflammation (3–5). The mentioned variations of energy metabolism can be performed in normoxic and hypoxic conditions (6, 7). Succinate has been found as a major metabolite accumulating in this procedure, as well as a novel extracellular inflammatory driver, which can be sensed extracellularly by succinate receptor 1 (Sucnr1) (8). Intracellular succinate in macrophages has been confirmed to stabilize hypoxia-inducible factor 1α (Hif-1α) for facilitating enhanced pro-inflammatory IL-1β production in the normoxic condition, since it can replace the function of inhibiting prolyl hydroxylase domain (PHD) enzyme activity under hypoxia (9). For this reason, extracellular succinate locally activates Sucnr1-expressing macrophages to initiate or intensify the immune response.

As confirmed by existing studies, succinate is abundantly detected in two immune diseases, i.e., synovial fluid (SF) of rheumatoid arthritis (RA) (3, 10) and serum of Crohn’s disease (11, 12). To be specific, the former has been proven as the upstream of IL-1β in SF. Endothelial cells, the inherent barrier of the vascular wall, are capable of releasing numerous inflammatory mediators and interacting with macrophages for amplifying inflammatory responses (13–15). However, the initial interaction between endothelial and macrophage remains unclear. On that basis, this study hypothesized that macrophages can locally deposit and release succinate after vascular endothelial injury, and then the accumulating succinate promotes vascular endothelial cells to impact the inflammatory response *via* succinate/Hif-1α/IL-1β signaling axis and facilitate the pathological characteristics of atherosclerosis.

## Materials and Methods

### Human Samples

All volunteers originated from cardiovascular clinics and wards of Shandong Provincial Qianfoshan Hospital. All of the volunteers signed informed consent forms. This study, complying with all the requirements of the Declaration of Helsinki, was reviewed and approved by the Ethics and Research Committee of the First Affiliated Hospital of Shandong First Medical University. Radial arterial blood was collected before coronary angiography. Subsequently, the volunteers fell into the positive (CHD group, >50%, n = 34) and negative groups (HC group, n = 38) in accordance with the tested degrees of coronary arteriostenosis. The blood was centrifugated at 3,000 rpm at 4°C for 10 min, and the serum was collected and kept at -80°C. **Supplementary Table 1** presents the statistics of clinical parameters.

### Mice

All mice were applied by complying with Regulations on the Administration of Experimental Animals under the promulgation and implementation of the State Science and Technology Commission. Beijing Vital River Laboratory Animal Technology Co., Ltd., offered Apoe^-/-^ and C57BL/6J mice, male, overall aged 8 weeks. C57BL/6J mice took the ordinary diet as the control (CON, n = 12), while Apoe^-/-^ mice (n = 26) had the western high-fat diet. After 12 weeks, 2 mice were randomly chosen to prove the formation of atherosclerosis plaque. After the model was made, Apoe^-/-^ mice were randomly divided into Apoe^-/-^ group (n = 12) and Apoe^-/-^+Suc group (n = 12). Succinate (0.039 mg/kg) in 0.9% normal saline (NS) was injected into Apoe^-/-^+Suc group mice once every other day (16), while 0.9% NS was injected into CON and Apoe^-/-^ group for comparison. The total of 7 injections lasted for 14 days. After another 7 feeding days, the mice were overall anesthetized with 10% hydrated chlorine aldehyde and perfused through the left ventricle with 0.9% NS. The blood sample was collected from cardiac apex and then centrifuged at 2,500 rpm for 5 min. Next, the serum was collected for ELISA. For the respective mice, the artery from heart to iliac artery was dissected and kept. Several fresh specimens were stored at -80°C to perform Western blots, and the others were fixed with 4% paraformaldehyde at the ambient temperature for 24 h. For the fixed specimens, the artery from aorta root to the iliac artery was stained with Oil-Red. The heart was embedded in optimal cutting temperature (OCT), and the aortic sinus was sectioned into 10-μm slices to perform Oil-Red, Masson, and H&E stain. Besides, the heart was embedded in paraffin, and the aortic sinus was sectioned into 6-μm slices to perform immunohistochemistry (IHC).

### Cell Culture and Treatments

Macrophage cell was differentiated from human monocyte THP-1 cell. THP-1 cell line was purchased from National Collection of Authenticated Cell Culture (ATCC) and then cultured within RPMI-1640 medium supplemented with 10% fatal bovine serun (FBS) using the incubator at 37°C with 5% CO_2_. Passages 10–15 were applied to perform the subsequent experiment. THP-1 cells were differentiated by employing 100 ng/ml phorbol 12-myristate 13-acetate (PMA; Sigma-Aldrich p8139#) for 24 h to obtain M0, and M0 is induced to M1 through Lipopolysaccharide (LPS) (100 ng/ml 24 h) stimulation. Since M1 expressed IL-1β, IL-6, tumor necrosis factor (TNF)-α, C-X-C motif ligand 8 (CXCL8), and IL-12B, rt-PCR was performed to verify the successful induction of M1 (17) (**Supplementary Figure 3**). Non-adherent macrophages were cleaned using phosphate buffered solution (PBS), and the adherent cells were cultured using fresh RPMI-1640 medium.

Human umbilical vein endothelial cells (HUVECs) were purchased from Sciencell and then maintained in Endothelial Cell Medium (ECM) media (Sciencell, USA) at 37°C using 5% CO_2_. All HUVECs used here were between passages 3 and 7.

Macrophages and HUVECs were counted by employing the hemocytometer and subsequently seeded in 6-, 12-, or 96-well plates to conduct the next study. Here, 0.25% trypsin-EDTA (Gibco, Canada) digested cells, and Serum-Free Cell Freezing Medium (NCM, China) cryopreserved cells. LPS (Sigma-Aldrich), PMA, and succinate were dissolved in PBS solution, and NF-56-EJ40 (MCE) was dissolved in Dimethyl Sulfoxide (DMSO). In the analysis of the pro-inflammatory function, cells were seeded in well plates added with LPS (100 ng/ml), succinate (800 μM) (3), or NF-56-EJ40 (4 μM) in accordance with the demand.

### Cell Counting Kit 8 Assay

The Cell Counting Kit 8 (CCK8) assay was performed for measuring the cell viability by complying with the manufacturer’s protocols (Beyotime, China). Macrophage cells were seeded in 96-well plate under a density of 10^4^ per well and adhering for 24 h. Next, the cell medium was changed to fresh medium supplemented with succinate or PBS, NF-56-EJ40, or DMSO. Following the experiment design, the cell activity was examined at 24 and 48 h of the incubation. The OD 450 absorbance was measured for the assessment of the cell activity. HUVECs’ seeded density reached 5,000 per well, and the remaining steps complied with those of macrophages.

### Hypoxia-Inducible Factor-1α Silencing

Macrophages and HUVECs were transiently transfected with NC siRNA or targeted Hif-1α silencer siRNA (20 nM for macrophages and 10 nM for HUVECs) in the presence of ribo FECT transfection reagent (Ribobio, China) for 36 h. The sequence of siRNA used is as follows: Sense GGAACCUGAUGCUUUAACUt and anti-sense AGUUAAAGCAUCAGGUUCCtt.

### ELISA

#### Succinate Detection

The levels of succinate in the human serum were measured by performing ELISA (Y-S Biotechnology, China) in accordance with the manufacturer’s instructions. The assay sensitivity reached 2.5 ng/L.

#### Interleukin-1β Detection

The levels of IL-1β in human serum and cell condition medium were examined by performing ELISA (BOSTER, China). The assay sensitivity was 1.95 pg/ml, and the OD 450 absorbance was examined to assess the indicators.

### Quantitative RT-PCR

Total RNA was extracted from cells with TRIzol (ambion by Life technologies, USA) method. Total RNA quantity was examined at 260 nm. cDNA was prepared with the use of the Reverse Transcription System (Bioer Technology, China) by performing SuperReal PreMix Plus assay (TIANGEN BIOTECH, China). ChemiDoc Imaging System (Bio-Rad) was employed to perform quantitative PCR. The results were determined based on the comparative Ct method. Primers of Sucnr1, Hif-1α, IL-1β, IL-6, TNF-α, CXCL8, IL-12B, and Glyceraldehyde-3-phosphate dehydrogenase (GAPDH) were presented below: Sucnr1-S, TATGGGATTGAGTTCGTTGTGG; Sucnr1-A, GAGAATGC TGGTATAGAGGTT GGC; Hif-1α-S, GCTCATCAGTTGCCACTTCCAC; Hif-1α-A, CCAAATCACCAGCATCCAGAAG; IL-1β-S, GGCTTATTACAGTGGCAATGAGG; IL-1β-A,GTAGT GGTGGTCGGAGATTCGT; GAPDH-S, GAAGGTGAAGGTCG GAGTC; GAPDH-A, GAAGATGGTGATGGGATTTC; TNF-α-S, CTGCCTFCAC TTTFFAG; TNF-α-A, ACATGGGCTACAGGCTTGTCACT; CXCL8-S, CACTGTG TGTAAACATGACTTCCAA; CXCL8-A, TGTGGTCCACTCTCAATCACTCTC; IL-12B-S, CTTGGAGCGAATGGGCATC; IL-12B-A, TGGGTCTATTCCGTTGT GTCTTTA. All the primers originated from Sangon Biotech (China).

### Western Blot

Total protein was extracted from cells or tissues with the use of RIPA Vs protease inhibitor cocktail (100:1), while cytoplasmic proteins and nucleoproteins were extracted by employing extraction kits (BOSTER, China). Protein concentration was quantified based on BCA kit (Beyotime, China). Equal proteins (15–25 μg) were separated on 10%–12% Sodium dodecyl sulfate (SDS) gels and then transferred onto Polyvinylidene fluoride (PVDF) (0.22/0.45 μm) membranes. After being blocked with 5% skim milk, the membranes were incubated with primary antibodies (1:1,000) overnight at 4°C. Primary antibodies consisted of Sucnr1 (Abcam, ab272856), Hif-1α (CST, #36169), IL-1β (CST, #12242), NLRP3 (Proteintech, #19771-1-ap), NF-κB (Proteintech, 66535-1-lg), Lamin B1 (Abcam, ab16048), Caspase-1 (Proteintech, 22915-1-ap), and β-Actin (Affinity, AF7018). Subsequently, the membranes were incubated with secondary antibody [goat anti-rabbit IgG HRP/goat anti-mouse IgG Horseradish Peroxidase (HRP) (1:10,000, ZSGP-BIO, China)] at Room temperature (RT) for 1 h. Bands were visualized with Enhanced chemiluminescence (ECL) reagent (MilliporeSigma, USA) and then captured on FluoChem E or ChemiDoc Imaging System. The optical density of bands was analyzed with the use of ImageJ software.

### Immunohistochemistry

Paraffin sections of the aortic sinus from mice were made for IHC after dewaxing and antigen repair. Subsequently, the sections were incubated with primary antibody (1:100) at 4°C overnight. The primary antibody included F4/80 (CST, #70076), CD31 (Affinity, AF6191), and antibodies in Western blot section. Next day, the respective section was incubated in secondary antibody at the ambient temperature for 30 min and then cleaned thoroughly with PBS. The tissues were stained with Diaminobenzidine (DAB) reagent and then observed under Leica DM1000 microscope. The images were investigated with ImageJ software.

### Statistical Analyses

For the clinical information, the values had the expression of mean ± SD. Continuous variables were determined by performing unpaired t-test, categorical variable was analyzed by performing χ^2^ test, and the correlation was explored by applying Pearson’s correlation coefficient. For *in vitro* and *in vivo* data, the values were expressed as mean ± SEM and then determined based on one-way ANOVA. *P* values <0.05 had statistical significance. Data were analyzed with GraphPad Prism 8.0 software.

## Results

### Expression of Succinate and Interleukin-1β in Atherosclerosis

**Figure 1:**
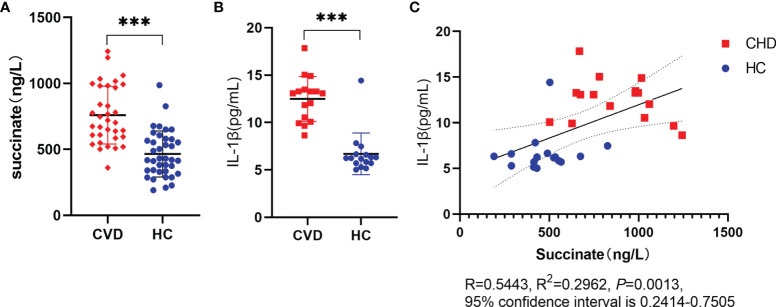
Expression of succinate and IL-1β in CHD. Radial arterial blood is collected before coronary angiography, then volunteers are divided into CHD group and HC group based on the results of coronary arteriostenosis. **(A, B)** Succinate and IL-1β were assessed *via* ELISA. Succinate detection (CHD group, n = 34; HC group, n = 38), IL-1β detection (CHD group, n = 16; HC group, n = 16), and all samples of this section had succinate detection. **(C)** Clinical correlation was analyzed in human samples using Pearson’s correlation coefficient (R = 0.5443, R^2^ = 0.2962, *P* = 0.0013, 95% confidence interval is 0.2414–0.7505). ***P < 0.001.

The clinical parameters were analyzed as **Supplementary Table S1**. CHD patients and the controls showed no difference between age, weight, Body Mass Index (BMI), gender, and smoking rate, whereas the two groups were noticeably different in alcohol drinking (*P* = 0.049). As reported by ELISA results of serum, succinate and IL-1β significantly increased in CHD patients in comparison with those in the HC group. Furthermore, Pearson’s correlation analysis was conducted on the two data, and results revealed a linear correlation between succinate and IL-1β in the two groups (R = 0.5443, R^2^ = 0.2962, 95% confidence interval is 0.2414–0.7505, *P* = 0.0013) (**Figures 1A–C**).

### Succinate Stimulates Human Umbilical Vein Endothelial Cells Producing Interleukin-1β

**Figure 2:**
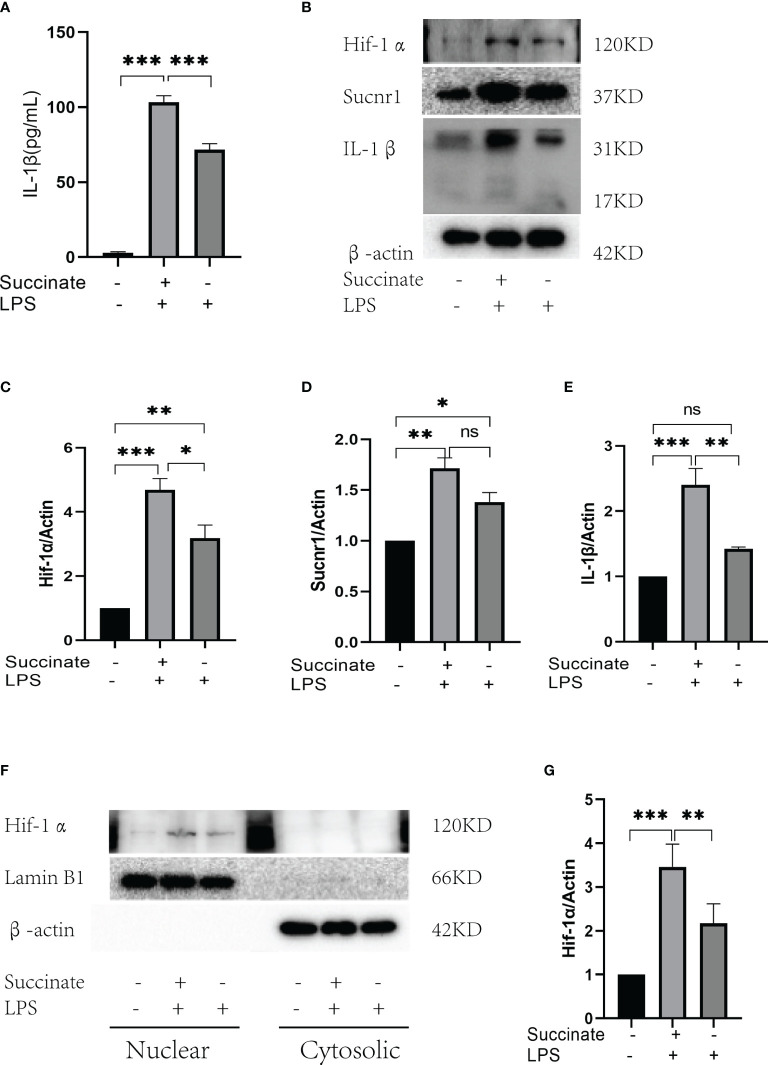
Sucnr1/Hif-1α/IL-1β signal axis in HUVECs. HUVECs were divided into three groups and given intervention according to design: Blank control was given equal amount of PBS, LPS+succinate group was given LPS (100 ng/ml) and succinate (800 μM), and LPS group was given LPS (100 ng/ml) for 24-h intervention, then supernatant medium and proteins were collected for experiment. **(A)** IL-1β of supernatant medium was detected *via* ELISA. **(B–E)** Expression levels of Sucnr1, Hif-1α, and IL-1β were displayed *via* Western blots and analyzed graphs. **(F, G)** Expression levels of Hif-1α in nucleus were detected using Western blots, in which Lamin B1 was the reference protein. Western blot data present mean ± SEM from three independent experiments at least. ELISA results represent mean ± SD from three different experiments at least. *P < 0.05; **P < 0.01; ***P < 0.001; ns means no statistical difference.

According to the verification result, succinate/IL-1β signaling axis existed in HUVECs. HUVECs were stimulated with LPS (100 ng/ml) and succinate (800 μM) as published literature. All drug doses of HUVECs here were the same as macrophages, and CCK8 was adopted to detect the toxicity of succinate to HUVEC, as shown in **Supplementary Figure 1**. Supernatant and holoprotein were extracted for Hif-1α and IL-1β detection among the dual stimulation group (LPS and succinate), the LPS group, and the blank control group. According to the result of ELISA, IL-1β of supernatant notably increased in the dual stimulation group (**Figure 2A**), and Western blots showed a similar result that Hif-1α and IL-1β were elevated under LPS and succinate stimulation (**Figures 2B–E**). Moreover, nuclear protein was extracted, and the results indicated that the expression level of Hif-1α in HUVEC nucleus was more significant under dual stimulation, which confirmed that succinate stimulation upregulated the expression of Hif-1α in HUVEC nucleus and facilitated its function as a transcription factor (**Figures 2F, G****)**.

### Sucnr1 Antagonist Inhibits Succinate/Interleukin-1β Signal Axis in Human Umbilical Vein Endothelial Cells and Macrophages

**Figure 3:**
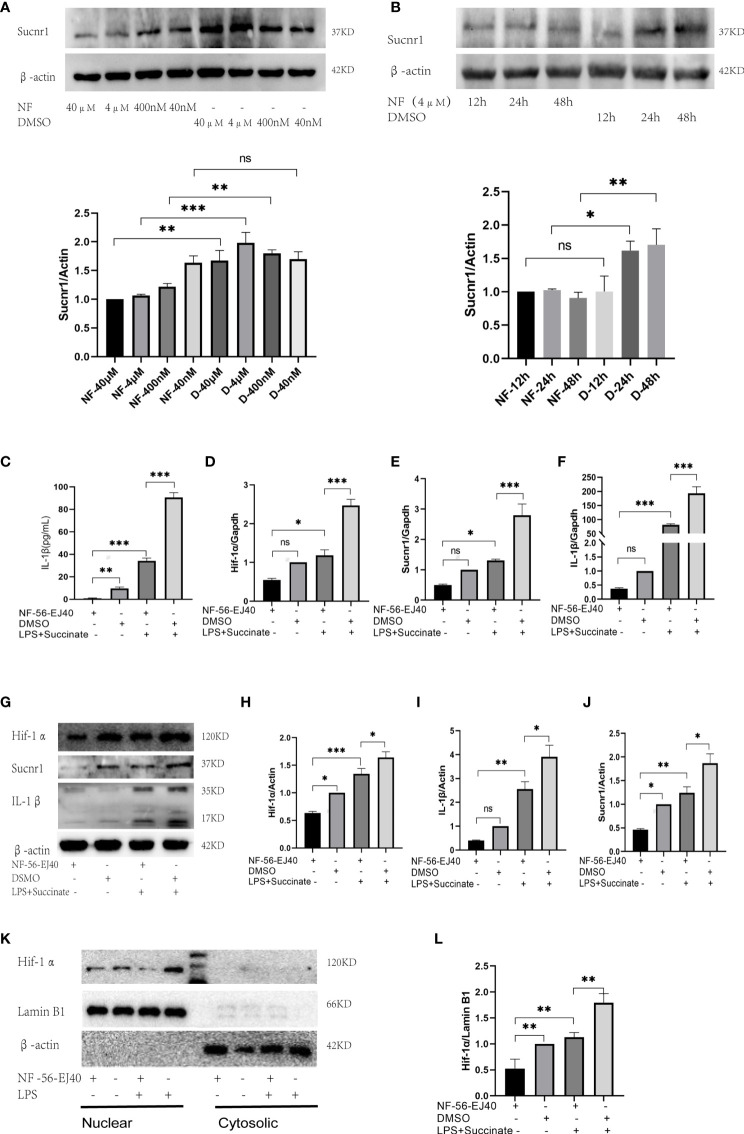
Sucnr1 antagonist inhibits succinate/IL-1β signal axis in HUVECs. **(A, B)** HUVECs were used as test cells to determine the optimum time and concentration of NF-56-EJ40 by Western blot. HUVECs were divided into four groups and given intervention according to design: NF-56-EJ40 group, DMSO group, NF-56-EJ40 and LPS+succinate group, DMSO and LPS+succinate group. **(C)** IL-1β of supernatant medium was detected *via* ELISA. **(D–F)** mRNAs of Sucnr1, Hif-1α, and IL-1β were tested using rt-PCR, in which Gapdh was the reference mRNA. **(G–J)** Expression levels of Sucnr1, Hif-1α, and IL-1β were displayed *via* Western blots. **(K, L)** Expression levels of Hif-1α in nucleus were detected using Western blots. Western blot data present mean ± SEM from three independent experiments at least. ELISA results represent mean ± SD from three different experiments at least. *P < 0.05; **P < 0.01; ***P < 0.001; ns means no statistical difference.

**Figure 4:**
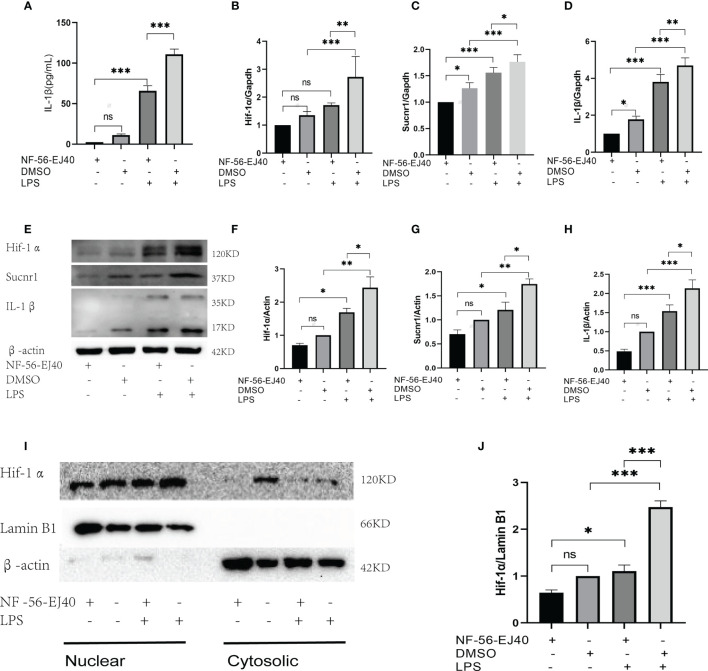
Sucnr1 antagonist inhibits succinate/IL-1β signal axis in macrophages. Macrophages were divided into four groups and given intervention according to design: NF-56-EJ40 group, DMSO group, NF-56-EJ40 and LPS group, DMSO and LPS group. **(A)** IL-1β of supernatant medium was detected *via* ELISA. **(B–D)** mRNAs of Sucnr1, Hif-1α, and IL-1β were tested using rt-PCR. **(E–H)** Expression levels of Sucnr1, Hif-1α, and IL-1β were displayed *via* Western blots. **(I, J)** Expression levels of Hif-1α in nucleus were detected using Western blots. Western blot data present mean ± SEM from three independent experiments at least. ELISA results represent mean ± SD from three different experiments at least. *P < 0.05; **P < 0.01; ***P < 0.001; ns means no statistical difference.

The succinate pathway was cut off to observe the expression of succinate downstream substances. As Sucnr1 is the membrane receptor of succinate, succinate should be combined with Sucnr1 to exert a pro-inflammatory activity. In this study, a high-affinity and highly selective antagonist (NF-56-EJ40, NF) was first adopted to interrupt Sucnr1 to cut off the signal axis. HUVECs served as the test cells to establish the concentration gradient and time gradient of NF intervention, and Sucnr1 levels were tested by Western blots (**Figures 3A, B****)**. The optimal concentration and time of NF-56-EJ40 intervention were 4 μM and 24 h. CCK8 results indicated that macrophage and HUVEC activities were not affected under the optimal concentration and time (**Supplementary Figure 1**). HUVECs were interfered with using NF for 24 h and LPS compared with succinate for the next 24 h. Sucnr1, Hif-1α, and IL-1β expression levels were tested using ELISA, rt-PCR, and Western blot (**Figures 3C–J**). Nuclear protein was also extracted to assess Hif-1α activation (**Figures 3K, L****)**. According to the results, NF-56-EJ40 could significantly inhibit Sucnr1 and lead to Hif-1α and IL-1β decreasing. In addition, the function of NF-56-EJ40 in macrophages was verified (**Figure 4**). As macrophages could produce succinate under LPS intervention, we only interfered it with NF-56-EJ40 4 μM for 24 h and then stimulated with LPS for another 24 h. All the results were similar as in HUVECs, which confirmed that NF-56-EJ40 could significantly reduce Hif-1α activation and IL-1β production through Sucnr1 inhibition in HUVECs and macrophages.

### The Significance of Hypoxia-Inducible Factor-1α in Succinate/Interleukin-1β Signal Axis

**Figure 5:**
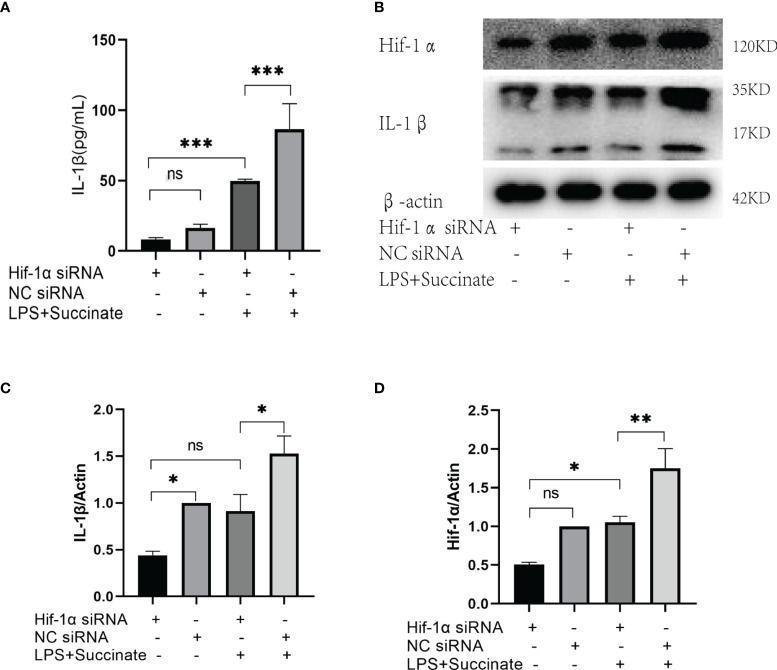
The expression of IL-1β after Hif-1α knockout. Hif-1α siRNA was transfected into HUVECs to downregulate Hif-1α as follows: Hif-1α siRNA group, NC siRNA group, Hif-1α siRNA and LPS+succinate group, NC siRNA and LPS+succinate group. **(A)** IL-1β of supernatant medium was detected *via* ELISA. **(B–D)** Expression levels of Hif-1α and IL-1β were assessed *via* Western blots. Western blot data present mean ± SEM from three independent experiments at least. ELISA results represent mean ± SD from three different experiments at least. *P < 0.05; **P < 0.01; ***P < 0.001; ns means no statistical difference.

As succinate can stimulate Hif-1α and IL-1β in macrophages and DCs in prior literature, we verified the significance and location of Hif-1α in HUVECs in this signal directly. Hif-1α siRNA was transiently transfected into HUVECs to downregulate Hif-1α, and the results indicated that the levels of Hif-1α and IL-1β decreased significantly in comparison with those in the NC siRNA group on the premise of succinate and LPS stimulation (**Figure 5**). Transfection efficiency of Hif-1α siRNA was verified by rt-PCR, as presented in **Supplementary Figure 2D**. All the results confirmed that in HUVECs, succinate upregulated the expression of inflammatory factor IL-1β by activating transcription factor Hif-1α.

### Nucleotide-Binding Oligomerization Domain(Nod)-Like Receptor 3 and Caspase-1 Are Involved in the Activation of Pro-Interleukin-1β Regulated by Hypoxia-Inducible Factor-1α

Referring to IL-1β, the NF-κB pathway cannot be bypassed. Thus, we explored whether there is a mutual influence between succinate/IL-1β signal axis and NF-κB pathway.

**Figure 6:**
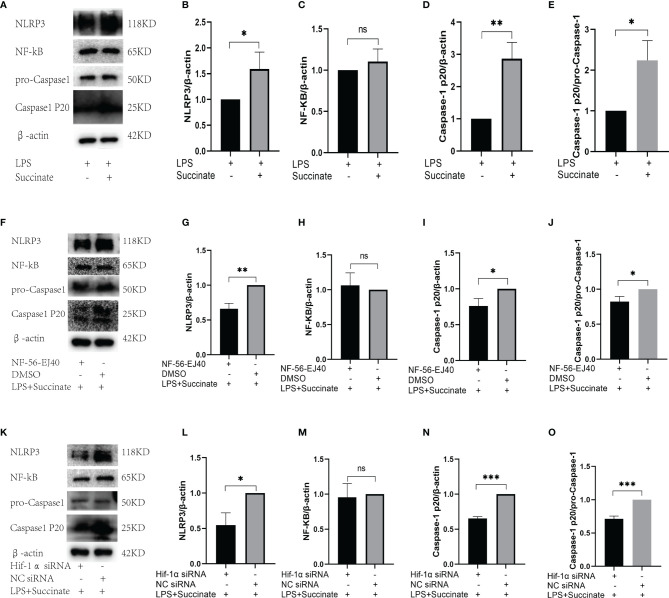
The relation between NF-κB pathway and succinate/IL-1β signal axis. **(A–E)** The expression levels of NF-κB, NLRP3, and Caspase-1 in HUVECs after succinate stimulation. **(F–J)** The expression levels of NF-κB, NLRP3, and Caspase-1 in HUVECs after NF-56-EJ40 inhibition. **(K–O)** The expression levels of NF-κB, NLRP3, and Caspase-1 in HUVECs after Hif-1α knockout. Western blot data present mean ± SEM from three independent experiments at least. *P < 0.05; **P < 0.01; ***P < 0.001; ns means no statistical difference.

Firstly, we tested whether succinate intervention affected protein expression in the NF-κB pathway. HUVECs were divided into two groups: one was LPS combined with succinate group; the other was LPS group. The stimulation lasted for 24 h, and NF-κB, NLRP3, and Caspase-1 were tested through Western blots. According to the results, NLRP3 and Caspase-1 notably increased after succinate stimulation, whereas the transcription factor NF-κB remained unchanged (**Figures 6A–E**).

Subsequently, we observed the effect of Sucnr1 inhibition on NF-κB pathway. Inhibitor (NF-56-EJ40) was used to interrupt Sucnr1 for 24 h, and then LPS and succinate were added for another 24 h. Western blots displayed that NLRP3 and Caspase-1 markedly decreased compared with those in the non-inhibitor group, whereas NF-κB had no obvious change (**Figures 6F–J**), which demonstrated that the two proteins were the downstream of succinate, and their location should be investigated during this axis.

Finally, we further examined whether this change occurred in Hif-1α/IL-1β section. Again, we silenced Hif-1α in HUVECs and intervened with LPS and succinate for 24 h. The expression levels of NLRP3 and Caspase-1 decreased compared with those in the NC siRNA group, and Hif-1α silencing did not affect NF-κB (**Figures 6K–O**), which demonstrated NLRP3 and Caspase-1 as the downstream of transcription factor Hif-1α and the upstream of activating IL-1β.

### Expression of Succinate/Interleukin-1β Signal in Human Umbilical Vein Endothelial Cells Cocultured With Macrophages

**Figure 7:**
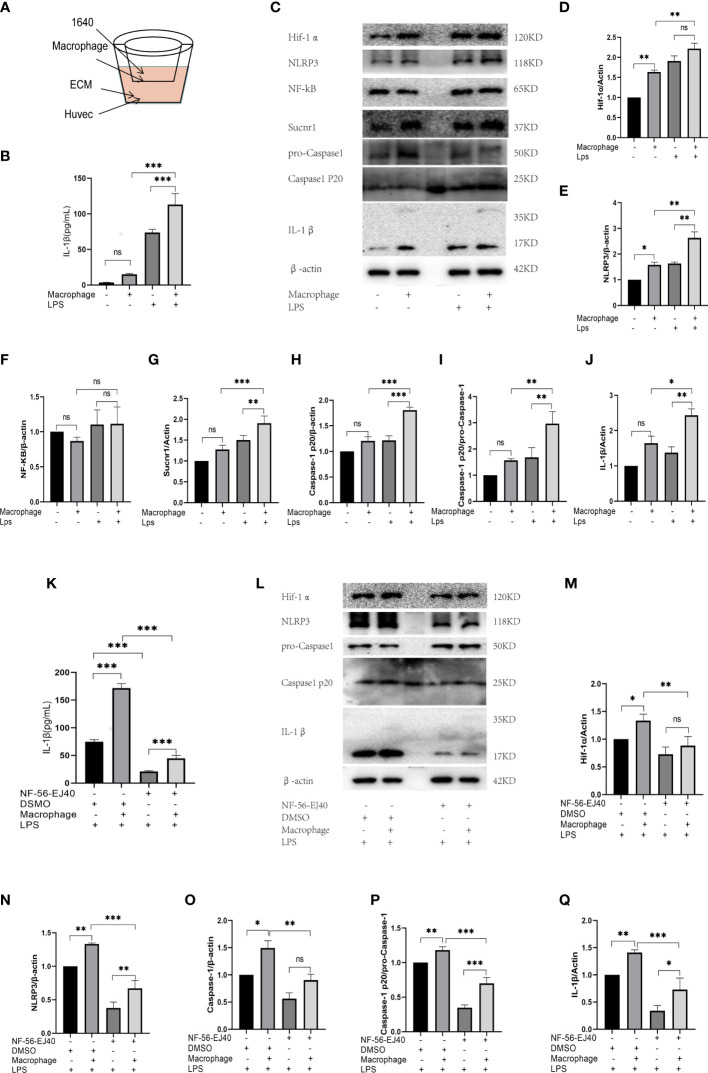
The expression of succinate/IL-1β signal axis during coculture system. **(A)** HUVECs were cocultured with macrophages in Transwell system, as shown in the figure. HUVECs were seeded in the down plate with 2.5 ml ECM complete medium, and macrophages were seeded in the upper fibrous membranes with 1.6 ml RPMI-1640 complete medium. The groupings of the coculture system were designed as follows: HUVECs with coculture medium (ECM and RPMI-1640), HUVECs cocultured with macrophages, HUVECs with coculture medium and LPS, HUVECs cocultured with macrophages and LPS, then the supernatant medium and holoprotein of HUVECs were extracted. **(B)** IL-1β of supernatant medium was detected *via* ELISA. **(C–J)** Expression levels of succinate/IL-1β signal axis and NF-κB were tested *via* Western blots. NF-56-EJ40 tried to rescue the inflammatory response of coculture system, and HUVECs were grouped as follows: I: LPS stimulated; II: cocultured with macrophages and LPS stimulated; III: NF-56-EJ40 inhibited and LPS stimulated; IV: NF-56-EJ40 inhibited, cocultured with macrophages, and LPS stimulated. Similarly, the supernatant medium and holoprotein of HUVECs were extracted. **(K)** IL-1β of supernatant medium was detected *via* ELISA. **(L–Q)** Expression levels of succinate/IL-1β signal axis and NF-κB were tested *via* Western blots. Western blot data present mean ± SEM from three independent experiments at least. ELISA results represent mean ± SD from three different experiments at least. *P < 0.05; **P < 0.01; ***P < 0.001; ns means no statistical difference.

In abnormal vessels, macrophages generally act as inflammatory trigger cells to affect endothelium, and together they exacerbate the immune inflammatory response. Thus, in this study, HUVECs were cocultured with macrophages given LPS stimulation to evaluate HUVEC inflammatory response in succinate/IL-1β signal axis. The coculture environment is presented in **Figure 7A**.

First, HUVECs were stimulated with LPS in the presence or absence of macrophages for 24 h, and then total protein of HUVEC was extracted. All the proteins of succinate/IL-1β signal axis were assessed by Western blots, and IL-1β of coculture supernatant was detected by ELISA. The results (**Figures 7B–J**) displayed that M1 could stimulate succinate/IL-1β signal axis of HUVECs to produce more IL-1β, as LPS could activate M0 differentiating to M1, which led to the release of succinate. The trends of NLRP3 and Caspase-1 were found to be consistent with those of Hif-1α and IL-1β, and NF-κB had no change in either coculture or pro-inflammatory stimulation.

Subsequently, we used Sucnr1 inhibitors to rescue the inflammatory process. HUVECs were divided into four groups (I: LPS stimulated; II: cocultured with macrophages and LPS stimulated; III: NF-56-EJ40 inhibited and LPS stimulated; IV: NF-56-EJ40 inhibited, cocultured with macrophages, and LPS stimulated). After intervention, total protein of HUVECs was extracted and supernatant was collected. As indicated from the result of Western blots, NF-56-EJ40 could significantly downregulate the expressions of Hif-1α, NLRP3, Caspase-1, and IL-1β compared with those in the non-inhibitor intervention group, and ELISA proved that NF-56-EJ40 effectively reduced IL-1β production (**Figures 7K-Q**).

### Succinate/Interleukin-1β Signal Axis *In Vivo*

Next, the investigation was conducted on how succinate activates inflammatory responses to atherosclerosis *in vivo*. C57BL/6J mice had the normal diet, while Apoe^-/-^ had the Western diet for 12 weeks and were divided into 2 groups. Subsequently, intravenous succinate was injected into the Apoe^-/-^+Suc group, and 0.9% NS was injected into the Apoe^-/-^ group.

**Figure 8:**
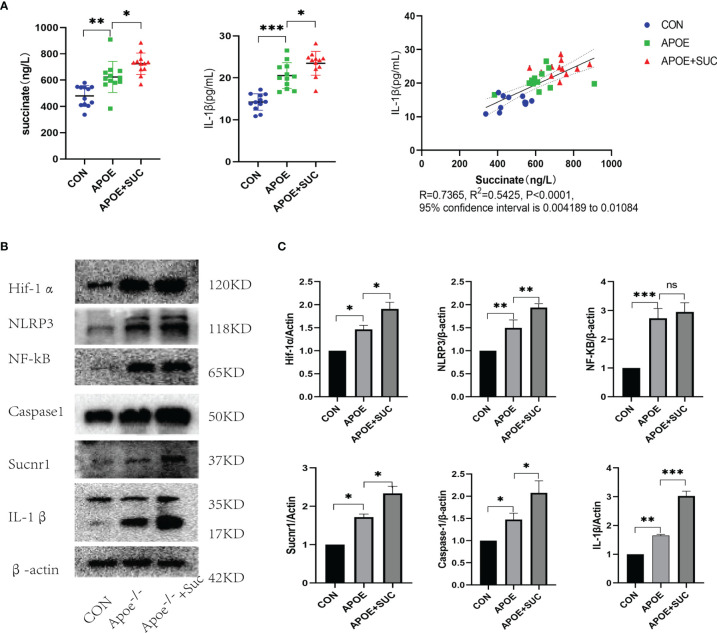
Succinate/IL-1β signal axis in atherosclerosis model (protein level). C57BL/6J mice were blank control (CON), while Apoe^-/-^ mice were randomly divided into two groups (Apoe^-/-^ group and Apoe^-/-^+Suc group). For each mouse, the artery from heart to iliac artery was dissected and kept. **(A)** Succinate and IL-1β of serum were detected by ELISA. ELISA results were mean ± SD from at least three different experiments. *P < 0.05; **P < 0.01; ***P < 0.001. **(B, C)** Expression levels of succinate/IL-1β signal axis and NF-κB were tested by Western blots. Western blot data present mean ± SEM from three independent experiments at least. *P < 0.05; **P < 0.01; ***P < 0.001; ns means no statistical difference.

As indicated by serum ELISA, succinate and IL-1β significantly increased in the Apoe^-/-^+Suc group compared with those in the Apoe^-/-^ group and CON group. Furthermore, Pearson’s correlation analysis was conducted on the two data, and the results showed that there was also a positive linear correlation between succinate and IL-1β (R = 0.7365, R^2^ = 0.5425, 95% confidence interval was 0.004189–0.01084, *P* < 0.0001) (**Figure 8A**). According to **Figures 8B, C**, the expressions of Hif-1α and IL-1β in the Apoe^-/-^+Suc group were largely upregulated in comparison with those in the Apoe^-/-^ group and CON group. Consistent with the above observation, after succinate injection, the expression levels of succinate/IL-1β signal axis including NLRP3 and Caspase-1 were mostly higher than those in the Apoe^-/-^ group and the Control (**Figures 8A, B****)**.

**Figure 9:**
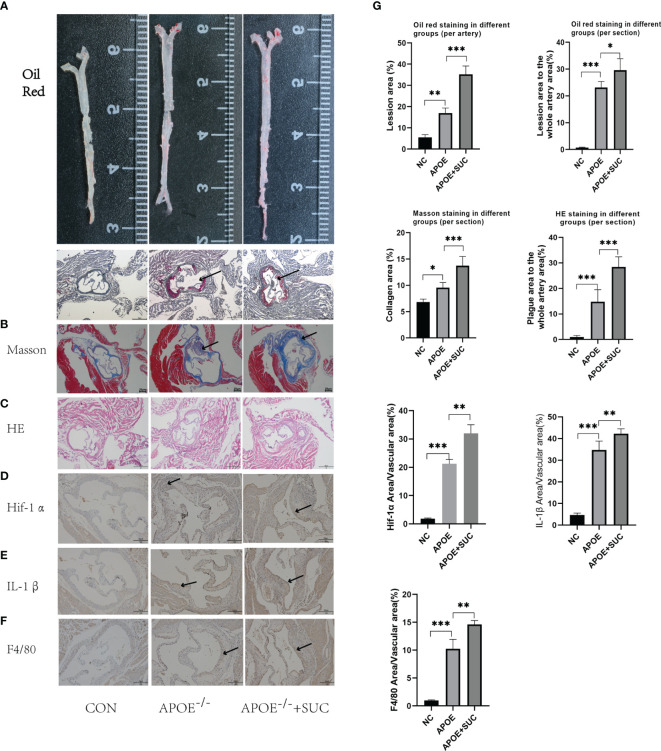
Succinate/IL-1β signal axis in atherosclerosis model (stainings). **(A, B)** Representative images of Oil Red O staining of the whole aorta (n = 3, per group) and aortic roots (n = 6–9, per group). **(C)** Representative images of H&E staining aortic roots (n = 6–9, per group). **(D)** Representative images of Masson staining aortic roots (n = 3–5). **(E–G)** Representative images of Hif-1α, IL-1β, and F4/80 staining aortic roots (n = 5–9). The areas of lesion or target proteins were marked with black arrows. **(A–C)** Magnification ×40; **(D–F)** magnification ×100. *P < 0.05; **P < 0.01; ***P < 0.001.

As revealed from the Oil Red, Masson, and H&E staining, succinate stimulation could accelerate the formation of atherosclerotic plaque and cause more severe fibrosis of wall and lumen narrowing (**Figures 9A–C, G**). Aortic root IHC was used to evaluate the inflammatory injured ability of succinate *in vivo*, and results indicated that M1 content (F4/80), Hif-1α, and IL-1β increased significantly in comparison with those of the two control groups (**Figures 9D–G**).

## Discussion

This study reported that the upregulation of serum IL-1β showed positive correlations with succinate in CHD patients. As indicated from in-depth study, succinate released by activated macrophages could drive the succinate/IL-1β signaling axis of endothelial cells and its own to amplify the intravascular inflammatory response and eventually could aggravate atherosclerotic inflammation.

Inflammation refers to the typical response of the host immune system to microbial infection or tissue injury (18, 19). However, excessive and unresolved inflammation generally causes detrimental chronic inflammatory diseases (3). Atherosclerosis refers to a cholesterol-induced inflammatory disease with macrophages and endothelial cells as the major protagonists (20, 21). During early atherogenesis, monocytes are recruited from the blood to the injured arterial vascular wall and locally differentiate into inflammatory macrophages (M1). Macrophages, inflammatory triggering cells, are capable of secreting considerable pro-inflammatory factors and stimulating the endothelium to impact the inflammatory response to amplify the inflammatory vascular injury (20). Since both types of cells exhibit pro-inflammatory functions, the sources of pro-inflammatory factors in existing studies could not be distinguished. Accordingly, how macrophages that activate endothelium causes a sustained inflammatory state in atherosclerosis remains unclear.

When macrophages are being activated, its energy metabolism will vary from oxidative phosphorylation to glycolysis concomitantly (22, 23). Succinate has been reported as a vital intermediate in glycolysis metabolism, which was demonstrated as a novel inflammatory signal in chronic inflammatory diseases [e.g., rheumatoid arthritis (3) as well as Crohn’s disease (12)]. Indeed, extracellular succinate increases because of local tissue ischemia or hypoxia (24), so both at the early stage of macrophage attachment to the endothelium and in the middle and late stages of plaque and thrombosis, excess succinate can be reasonably present in the pathological process of atherosclerosis.

This study first demonstrated that succinate significantly elevated in arterial serum in CHD patients compared with HC based on targeted detection, and the inflammatory marker IL-1β (2, 25) presented a consistent trend. Subsequently, as revealed by the Pearson correlation analysis, a positive relationship was found between the mentioned two materials. According to existing studies, succinate could induce IL-1β through Hif-1α in activated macrophages (19), whereas whether endothelial cell IL-1β is directly correlated with succinate increasing and whether it can act as a bridge between macrophages and endothelial cells to explain the internal mechanism of the enlarged inflammatory response should be explored in depth.

Succinate, an inflammatory signal, only has a conducting effect in the presence of Sucnr1. Sucnr1/GPR91 refers to a G protein-coupled cell surface sensor in terms of extracellular succinate (26–29), which is stably expressed on immature Dendritic cells (DCs) and macrophages in immune systems (28). As reported by the cell experiments, Sucnr1 could be stably expressed in HUVECs, thereby creating a pathway for uptaking external succinate. Subsequently, the data of this study indicated that exogenous succinate successfully activated Hif-1α and stabilized its nuclear expression in HUVECs, whereas the IL-1β level rose notably. However, when Hif-1α was disrupted, IL-1β production was significantly reduced in HUVECs. The mentioned results revealed that extracellular succinate alone led to a moderate induction of Hif-1α in HUVECs and significantly enhanced its transcription function, thereby ultimately potentiating IL-1β production.

Next, we demonstrated the necessity of Sucnr1 in this inflammatory signaling axis using an antagonist of Sucnr1 NF-56-EJ40. NF-56-EJ40 blocked succinate signal transduction and led to a reduction of Hif-1α production and transcription activity, thereby directly causing IL-1β production to decrease in HUVECs and macrophages. This result confirmed Sucnr1 as the only pathway that succinate mediated Hif-1α/IL-1β signal, and this study found the possibility of reducing the production of the inflammatory factor IL-1β by blocking Sucnr1.

However, the production of IL-1β is complicated, and NF-κB pathway is the most famous and classical one. Our previous results can only explain Hif-1α as a transcription factor that regulates pro-IL-1β production, whereas activated IL-1β should be generated in the presence of Caspase1, which serves as the major material of NF-κB pathway (1, 30, 31). We found that the downstream nodes of NF-κB pathway NLRP3 and Caspase-1 increased significantly in HUVECs after succinate stimulation, whereas the level of transcription factor NF-κB remained almost unchanged. Next, the NF-56-EJ40 intervention experiment displayed that NLRP3 and Caspase-1 notably decreased after the interruption of succinate transduction, while NF-κB was stable. The mentioned results suggested that succinate combined with Sucnr1 could activate NLRP3/Caspase-1 cascades, whereas it showed no correlation to their upstream NF-κB. Next, we further determined the position and origin of NLRP3/Caspase-1 in succinate signal axis. In the following study, the downregulation of Hif-1α *via* siRNA led to a decrease in NLRP3 production at baseline, thereby proving that Hif-1α is a transcription factor that could positively regulate NLRP3 gene synthesis. NLRP3 could activate and recruit adapters to form inflammasome complexes in response to inflammation or other imprints, then inflammasome joints to pro-caspase-1 (1, 30–32). Caspase-1 is activated by autoproteolysis and formation of the enzymatically active heterotetramer, and then it catalyzes pro-IL-1β cleavage to IL-1β (33–35).

**Figure 10:**
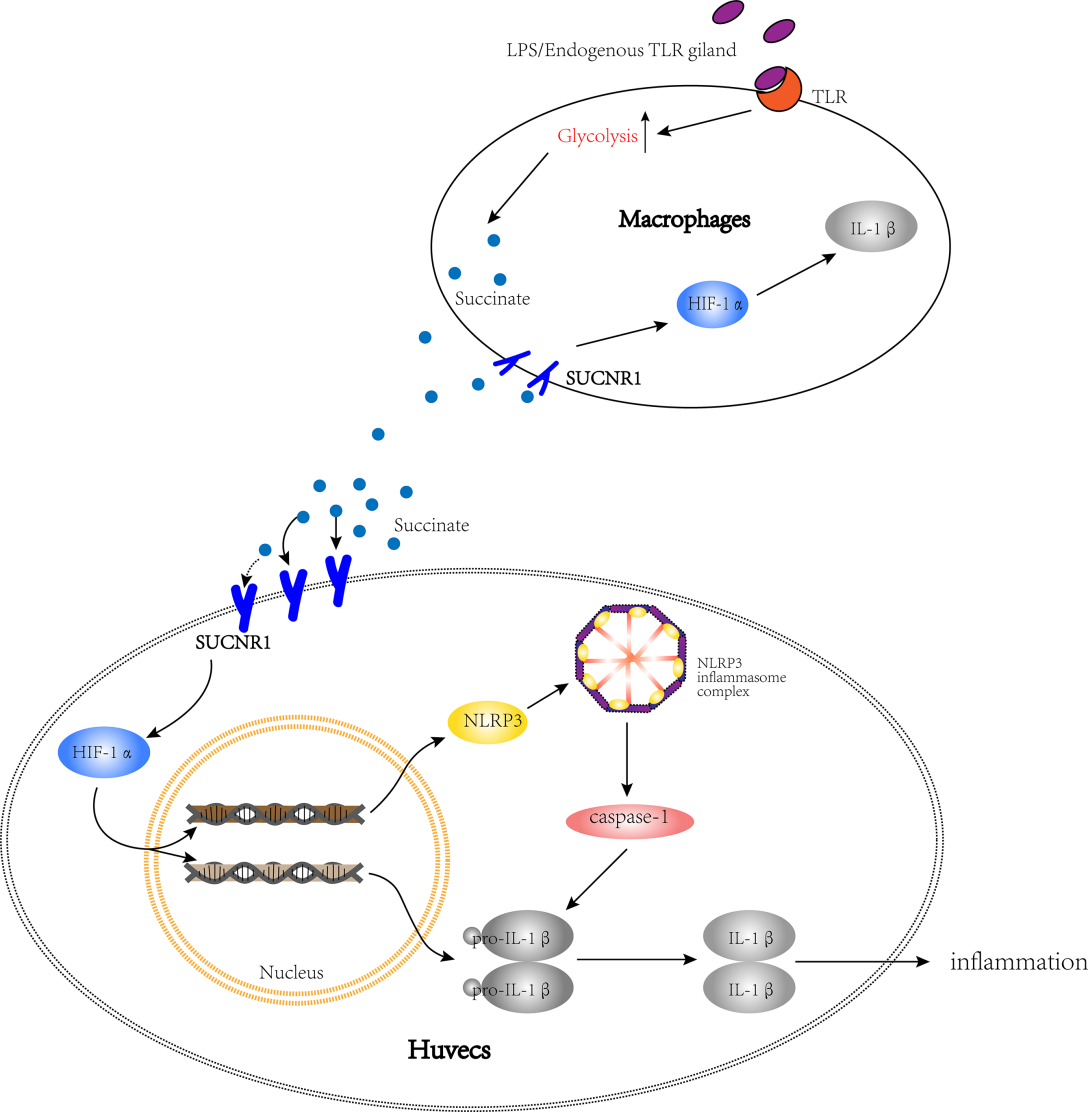
The path diagram of succinate/IL-1β signal axis between HUVECs and macrophages. The bigger cell is HUVEC, while the smaller one is macrophage. This figure shows that after being activated, macrophages release succinate and stimulate the succinate/IL-1β signal axis in HUVECs to promote an inflammatory response.

All of the above data were based on the increased succinate in vascular microenvironment, so we cocultured HUVECs with activated macrophages to verify the existence of succinate/IL-1β signal axis in HUVECs. The results confirmed that in injured vessel microenvironment, macrophages were converted into M1 and attached to the vascular endothelium, thereby releasing considerable succinate with the transformation of energy metabolism. Succinate could bind with Sucnr1 entering HUVECs and then activate the transcription factor Hif-1α to localize to the nucleus, which could induce the production of pro-IL-1β and NLRP3. NLRP3 is the major of NLRP3 inflammasome, which could link to pro-caspase-1 and release active caspase-1. Lastly, active caspase-1 catalyzed IL-1β processing by exacerbating the inflammatory cycle (**Figure 10**).

Based on the mentioned results, we also confirmed the pro-inflammatory effect of succinate *in vivo*. In atherosclerosis, external succinate can trigger the succinate/IL-1β signaling axis and intensify the inflammatory response, which leads to the thickening of atherosclerotic plaques, the fibrosis of artery wall, and the narrowing of the lumen. In this pathological mechanism, succinate stimulation was also reported to enhance NLRP3 and caspase-1 expression, whereas NF-κB was not visibly affected. The mentioned results confirmed that glycolysis only stabilized the activity of transcription factor Hif-1α, whereas the expression of NF-κB was not affected. Second, glycolysis can affect the assembly of NLRP3 inflammasome through Hif-1α and ultimately regulate IL-1β production.

In brief, glycolytic metabolism already exists when macrophages recruit and attach to vascular endothelium at the early stage of atherosclerosis, accompanied by succinate production. On the one hand, succinate enters the circulation. On the other hand, it enters the endothelium and macrophages and starts succinate/IL-1β signal axis to produce considerable inflammatory factors, thereby exacerbating the inflammatory process of atherosclerosis. According to cell studies, NF-56-EJ40 has been reported as an effective, high-affinity, and significantly selective human Sucnr1 antagonist, capable of interrupting succinate/IL-1β signal axis in endothelium and macrophages for the suppression of inflammation responses. Our study provides a point that energy metabolism switch can initiate an immune response to affect the surrounding tissue to exacerbate an inflammatory response. Sucnr1 may act as a novel target for cutting off succinate signal transduction to prevent the inflammatory process of atherosclerosis.

### Limitations

Since frozen slices are rare, immunofluorescence and co-location analysis of Hif-1α and IL-1β were not performed. Otherwise, Sucnr1 antagonist (NF-56-EJ40) is currently only available in human cells, and Sucnr1^-/-^/Apoe^-/-^ mice were not created to prove the significance of Sucnr1 *in vivo*. Subsequently, the significance of this signal axis for atherosclerosis will be further explored in animal experiments.

## Data Availability Statement

All data needed to evaluate the conclusions in the paper are present in the paper and/or the **Supplementary Material**. Further information and requests for resources and reagents should be directed to and will be fulfilled by the lead contact YH.

## Ethics Statement

The studies involving human participants were reviewed and approved by the Ethics and Research Committee of the First Affiliated Hospital of Shandong First Medical University. The patients/participants provided their written informed consent to participate in this study. The animal study was reviewed and approved by the Ethics and Research Committee of the First Affiliated Hospital of Shandong First Medical University.

## Author Contributions

JX and YH designed the research. JX performed most of the experiments and analyzed the data. YBZ, YQZ, YJZ, XW, HL, AZ, WW, and JW gave according guidance or support to the project. All authors contributed to the article and approved the submitted version.

## Funding

This work was supported by the National Natural Science Foundation of China (NSFC; no. 8197021024), Taishan Scholar Engineering Construction Fund of Shandong Province (no. ts201511104), Traditional Chinese Medicine Science and Technology Project of Shandong Province (no. 2020Q036), and National Natural Science Foundation of Qianfoshan Hospital, Shandong Province (no. QYPY2021NSFC0608).

## Conflict of Interest

The authors declare that the research was conducted in the absence of any commercial or financial relationships that could be construed as a potential conflict of interest.

## Publisher’s Note

All claims expressed in this article are solely those of the authors and do not necessarily represent those of their affiliated organizations, or those of the publisher, the editors and the reviewers. Any product that may be evaluated in this article, or claim that may be made by its manufacturer, is not guaranteed or endorsed by the publisher.

## Supplementary Material

Supplementary Table 1 Clinical parameter statistics.

Supplementary Figure 1 Cell viability assay for drug intervention *via* CCK8.

Supplementary Figure 2 Transfection efficiency of Hif-1α siRNA *via* rt-PCR.

Supplementary Figure 3 Expression of representative M1 marker genes of THP-1 macrophage after 24 h of LPS polarization.

Supplementary Figure 4 Raw data review incoming and outgoing emails.

Supplementary Figure 5 Repeated experiment of caspase-1 in a blot.

Supplementary Figure 6 Sucnr1 blots between DMSO and blank control.

Supplementary Figure 7 En face staining of Aortic endothelium.
