# Supplementary material for: Succinate/IL-1β Signaling Axis Promotes the Inflammatory Progression of Endothelial and Exacerbates Atherosclerosis
Source: Front Immunol. 2022 Feb 22;13:817572. doi: 10.3389/fimmu.2022.817572 (PMC8901997; doi:10.3389/fimmu.2022.817572)
Supplement: Supplementary Table 1 — Clinical parameter statistics. [file Table_1.docx]

| **Groups**  **Items** | **CHD** | **HC** | ***P* value** |
| --- | --- | --- | --- |
| **ages（Mean±SD）** | 61.47±10.22 | 62.8±8.56 | 0.6696 |
| **Sex (Male)** | 61.76% | 55.26% | 0.5764 |
| **Smoking** | 58.82% | 57.89% | 0.9364 |
| **Drinking** | 82.35% | 60.35% | 0.049  * |
