# Supplementary figures and images for: Succinate/IL-1β Signaling Axis Promotes the Inflammatory Progression of Endothelial and Exacerbates Atherosclerosis
Source: Front Immunol. 2022 Feb 22;13:817572. doi: 10.3389/fimmu.2022.817572 (PMC8901997; doi:10.3389/fimmu.2022.817572)

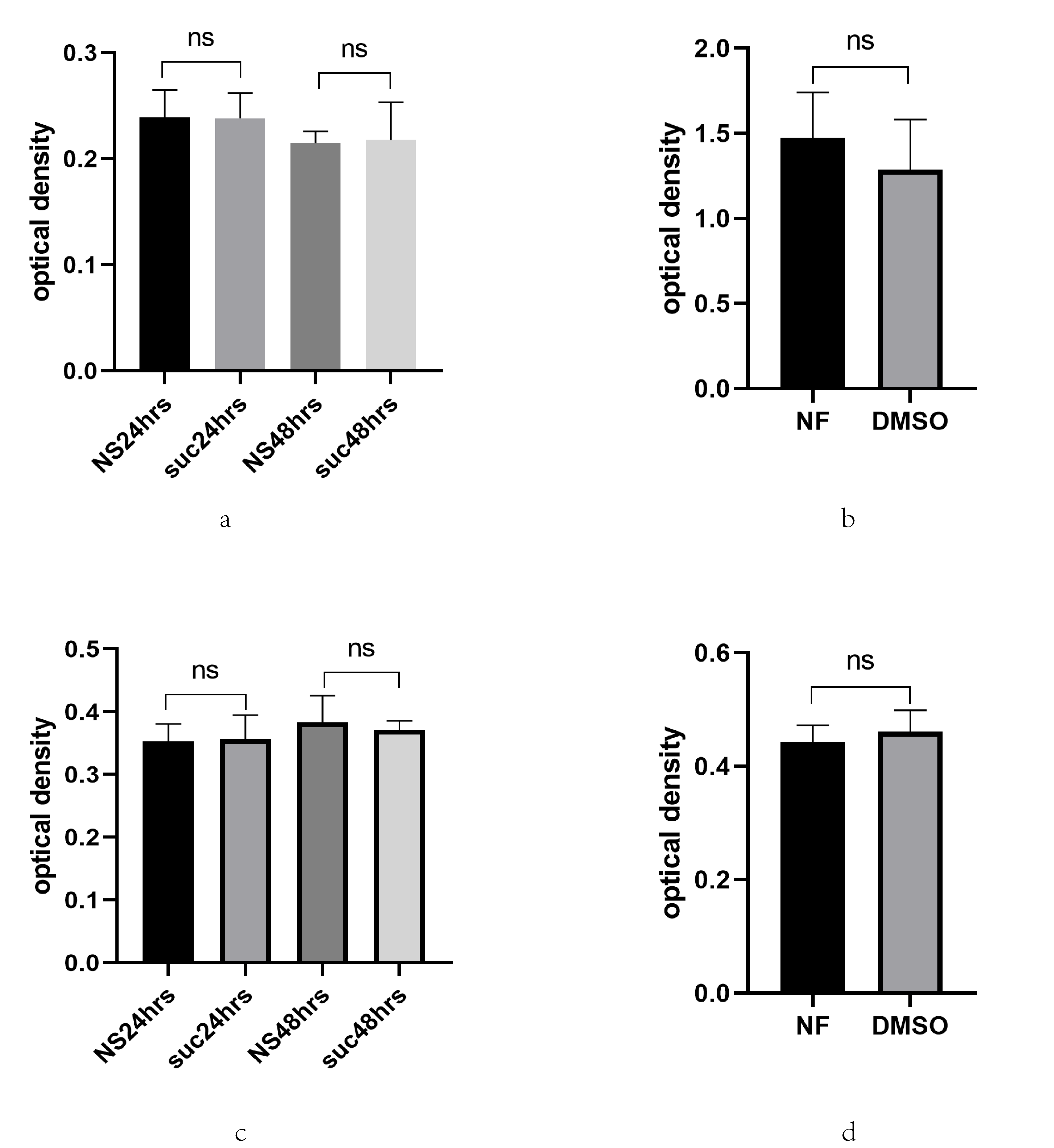

Supplement: Supplementary Figure 1 — Cell viability assay for drug intervention via CCK8. [file Image_1.tif]

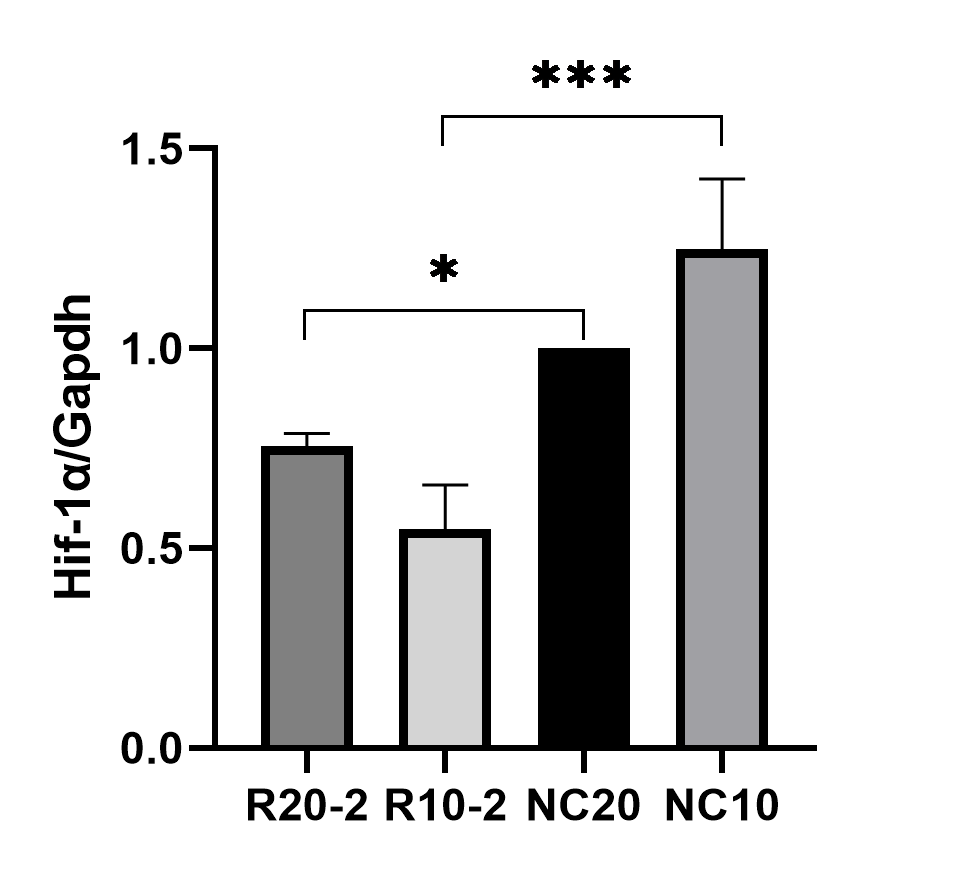

Supplement: Supplementary Figure 2 — Transfection efficiency of Hif-1α siRNA via rt-PCR. [file Image_2.tif]

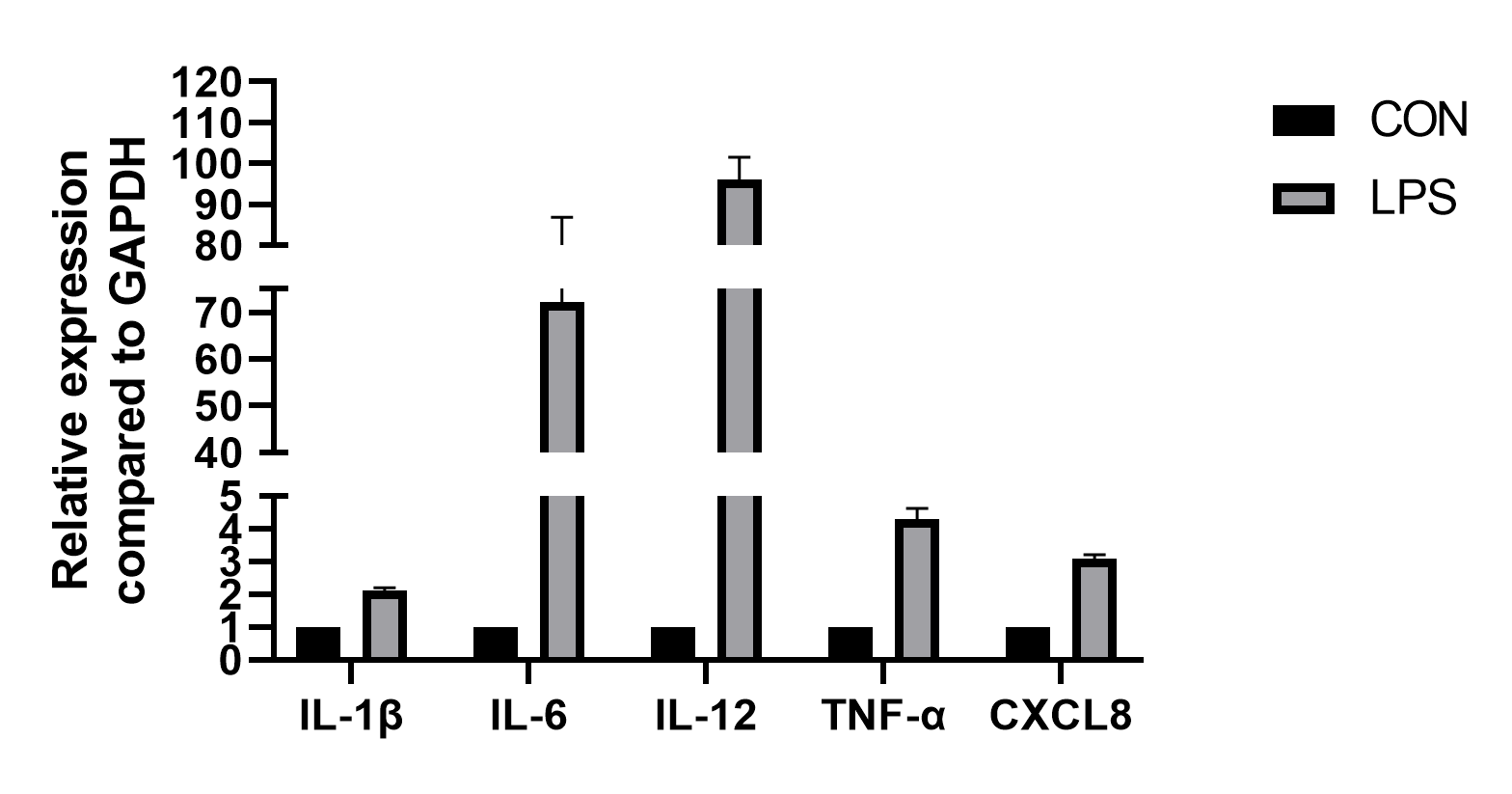

Supplement: Supplementary Figure 3 — Expression of representative M1 marker genes of THP-1 macrophage after 24 h of LPS polarization. [file Image_3.tif]

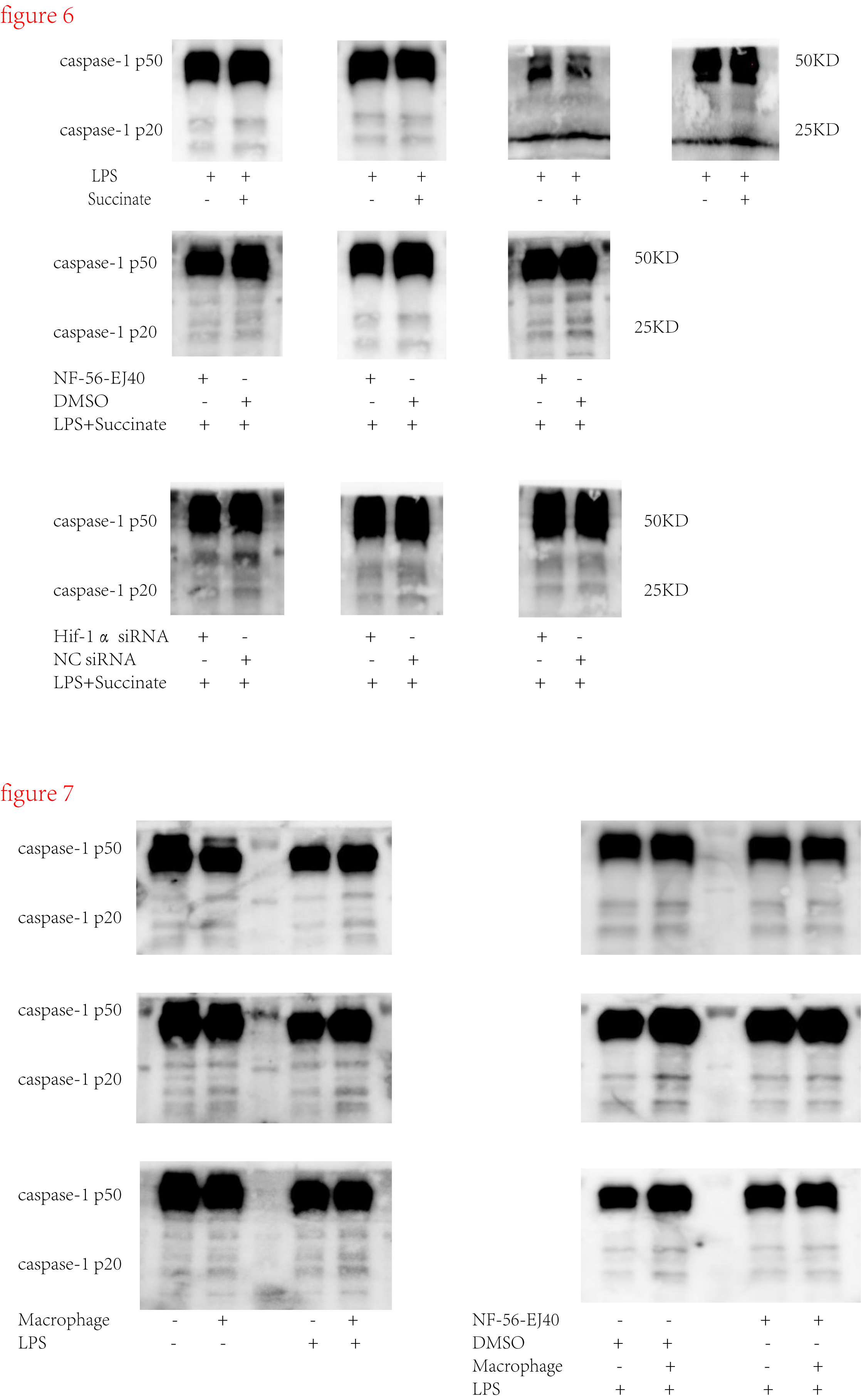

Supplement: Supplementary Figure 4 — Raw data review incoming and outgoing emails. [file Image_4.tif]

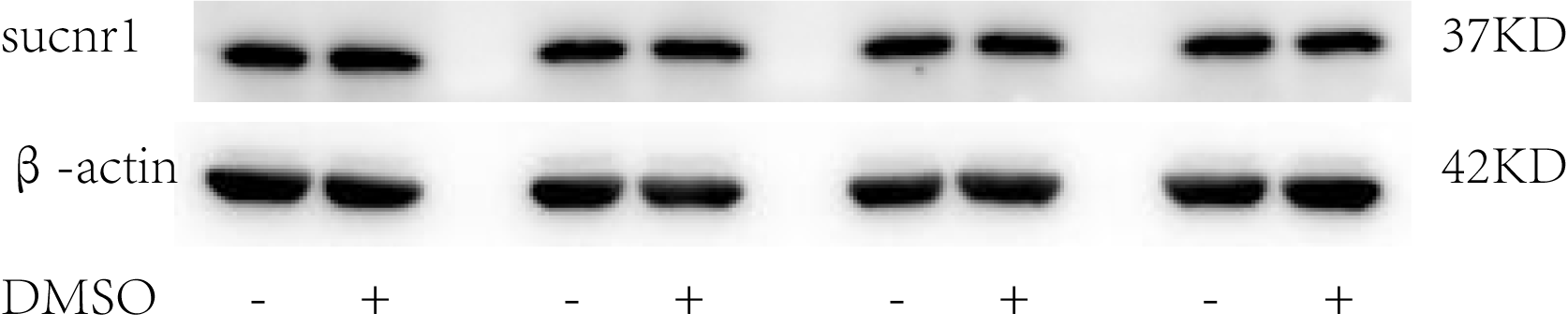

Supplement: Supplementary Figure 5 — Repeated experiment of caspase-1 in a blot. [file Image_5.tif]

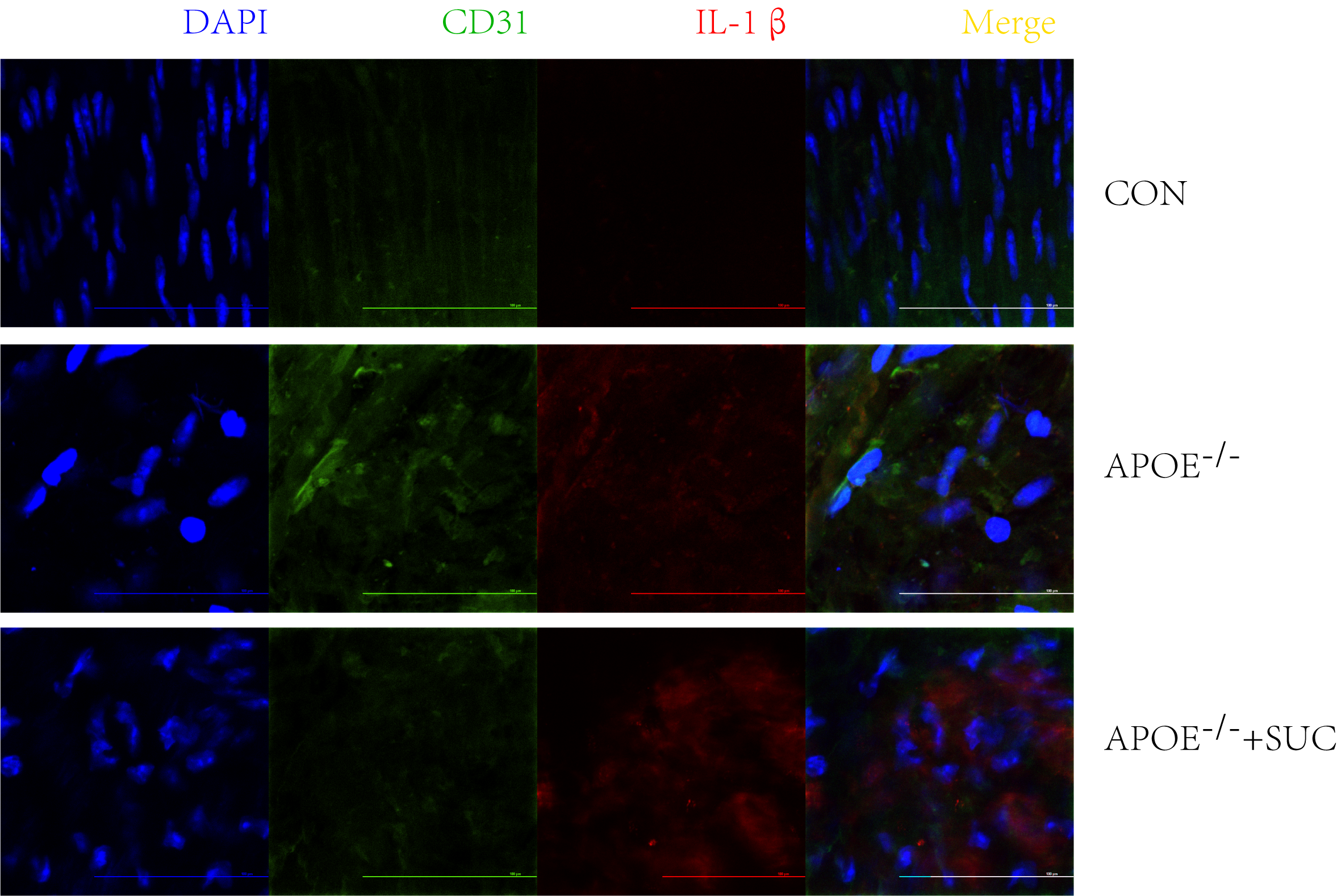

Supplement: Supplementary Figure 6 — Sucnr1 blots between DMSO and blank control. [file Image_6.tif]
